# Two autochthonous cases of Crimean-Congo haemorrhagic fever and the One Health response, Thessaly, Greece, 2025

**DOI:** 10.2807/1560-7917.ES.2025.30.50.2500717

**Published:** 2025-12-18

**Authors:** Danai Pervanidou, Sara Georgiadou, Elisavet Stavropoulou, Aggelos Stefos, Katerina Tsioka, Chrysovalantou Niki Kefaloudi, Nikolaos Gatselis, Konstantinos Makaritsis, Demosthenes Makris, Parisi Kyriaki, Sofia Chatzianastasiou, Antonios Maragkos, Theano Georgakopoulou, Dimitra Paraskeva, Dimitrios Paraskevis, Olga Papachristou, Styliani Papatheodorou, Spyridoula Damaskou, Varvara Kaouna, Smaragda Sotiraki, Anastasios Saratsis, Aggeliki Liakata, Dimitrios Papasteriou, Evangelos Kartsoulis, Zacharoula Bogogiannidou, Stamatia Kokkali, Ioanna Voulgaridi, Styliani Sarrou, Konstantina Stoikou, Styliani Pappa, Ourania Tsakalidou, Varvara Mouchtouri, Katerina Marinou, Ilektra A Fragkou, Dimitrios Hatzigeorgiou, Anna Papa, George N Dalekos, Christos Hadjichristodoulou

**Affiliations:** 1 National Public Health Organisation, Athens, Greece; 2 European Reference Network on Hepatological Diseases (ERN RARE-LIVER), Department of Medicine and Research Laboratory of Internal Medicine, National Expertise Center of Greece in Autoimmune Liver Diseases, General University Hospital of Larissa, Larissa, Greece; 3 National Reference Centre for Arboviruses and Haemorrhagic Fever viruses, Aristotle University of Thessaloniki, Thessaloniki, Greece; 4 Department of Intensive Care, School of Medicine, Faculty of Health Sciences, University of Thessaly, Larissa, Greece; 5 Department of Hygiene, Epidemiology and Medical Statistics, School of Medicine, National and Kapodistrian University of Athens, Athens, Greece; 6 Department of Infection Prevention and Control, General University Hospital of Larissa, Larissa, Greece; 7 Veterinary Research Institute Hellenic Agricultural Organization (ELGO – DIMITRA), Thessaloniki, Greece; 8 Regional Veterinary Authorities, Region of Thessaly, Larissa, Greece; 9 Laboratory of Hygiene and Epidemiology, Faculty of Medicine, University of Thessaly, Larissa, Greece; 10 Department of Immunology and Histocompatibility, Faculty of Medicine, University of Thessaly, Larissa, Greece; 11 Ministry of Rural Development and Food, Directorate General of Veterinary Services, Directorate of Animal Health, Athens, Greece; * These authors contributed equally to this work and share first authorship.; ** These authors contributed equally to this work and share last authorship.

**Keywords:** Crimean-Congo Haemorrhagic fever, autochthonous, Greece, nosocomial infection, healthcare worker, tick, One Health, zoonosis, tick-borne

## Abstract

In June 2025, two autochthonous Crimean-Congo haemorrhagic fever cases were recorded in Greece; a fatal index case and a healthcare worker secondarily infected. With only one autochthonous case previously reported in Greece, in 2008, this event was unexpected and triggered a One Health response: cases investigation, contact tracing, infection prevention and control guidance, field investigation, preventive measures targeting vectors and possible animal hosts, as well as awareness-raising measures. Although Greece is non-endemic for Crimean-Congo haemorrhagic fever, some neighbouring countries are endemic, and this event underscores the need for enhanced surveillance, vigilance and multisectoral collaboration.

**Key public health message**

**What did you want to address in this study and why?**

Crimean-Congo haemorrhagic fever (CCHF) is a severe viral haemorrhagic disease transmitted by ticks. We report the second case ever locally acquired in Greece, which was fatal. This case led to an infection in a healthcare worker, who recovered. This unexpected event occurred in a country where this disease is not considered to be established.

**What have we learnt from this study?**

The occurrence of CCHF in different regions may shift over time as climate change influences vector-borne diseases. A high index of suspicion should be maintained when the clinical presentation is compatible. Timely outbreak investigations and control measures remain crucial in containment and should also involve the veterinary and the environmental sector (One Health approach).

**What are the implications of your findings for public health?**

This event highlights the need for multisectoral collaboration across human, animal, and environmental health sectors. Enhanced surveillance, healthcare preparedness, and public awareness are essential to prevent and rapidly respond to future threats.

## Background

Crimean-Congo haemorrhagic fever (CCHF) is an acute, often severe viral haemorrhagic disease caused by the CCHF virus (CCHFV), a member of the genus *Orthonairovirus* [1]. It is a tick-borne zoonosis, transmitted primarily through bites of infected *Hyalomma* ticks (the principal vector) or through direct contact with blood or bodily fluids of infected patients and animals. Clinical manifestations typically include fever, myalgia and headache, with severe cases progressing to haemorrhage and multi-organ failure [1]. The geographical distribution of CCHF is broad, it is endemic in Africa, the Middle East (including Türkiye), south-east Asia, and parts of eastern Europe (some Balkan countries) [2,3]. Also in Spain, local transmission events have been recorded since 2013 (on an annual basis since 2020) [4] and unexpected cases have sporadically been reported in non-endemic countries such as Greece, where one autochthonous case was recorded in 2008 [5].

Healthcare-associated transmission remains a critical concern; breaches in standard precautions can lead to nosocomial clusters, as seen in several countries [6-8]. When personal protective equipment (PPE) is inadequate, secondary human-to-human spread can occur via direct contact with blood or tissues of viraemic patients [9]. 

## Outbreak detection

On 25 June 2025, the National Reference Centre (NRC) for Arboviruses and Haemorrhagic Fever viruses notified a case of CCHF to the National Public Health Organisation (NPHO) in Greece. The case concerned an older adult (> 65 year), with a history of urinary tract malignancy, who was admitted to hospital because of a history of 2 days of high fever (39.5 °C), rigors, abdominal pain, vomiting and persistent frontal headache. The patient resided in a rural village (altitude ca 500 m above sea level), and was regularly visiting an animal farm, but had no history of recent travel. On initial evaluation, the patient was lethargic with high fever, tachycardia (110 bpm) and macroscopic haematuria. Laboratory tests revealed anaemia, notable thrombocytopenia, prolonged prothrombin and partial thromboplastin times, low fibrinogen, abnormal liver enzymes, extremely high ferritin levels and acute kidney injury. Multiple sets of blood and urine cultures were performed, and the patient was started immediately on broad spectrum antibiotic treatment for suspected urinary tract infection. Gradually, the patient developed severe thrombocytopenia (platelets 6,000/μL; normal range: 150,000–450,000/μL) with progressively increasing levels of lactate dehydrogenase (LDH), ferritin, and deteriorating renal function. We suspected CCHF and blood samples were sent to the NRC, confirming diagnosis and triggering response.

Here, we report two autochthonous CCHF cases occurring in Greece in 2025 with distinct transmission pathways and describe the associated public health and One Health responses.

## Methods

### Definition of cases and contacts

We used the European Union case definition for viral haemorrhagic fevers [12]. A contact was defined as a person directly or indirectly exposed to blood, body fluids or tissues of a viraemic patient.

### Epidemiological, epizootiological and environmental investigations

The NPHO and the hospital infection control committee conducted case investigation and contact tracing. National and regional veterinary authorities and ELGO Dimitra researchers conducted targeted field epizootiological investigations, including animal blood sampling and tick collection from animals and environment, at the sheep farm the patient had regularly visited. In addition, we assessed national tick surveillance data (maintained through the EU4HEALTH European project OH SURVector), to collect available information on the distribution and abundance of the vector in the broader area. Wildlife was not targeted for investigation. 

### Microbiological investigations

#### Serological methods

For the detection of CCHFV IgM and IgG antibodies in humans, we used the VectoCrimean – CHF-IgM and VectoCrimean – CHF-IgG ELISA kits (Vector-Best, Russia). For the detection of antibodies against CCHFV in animals, we used the ID Screen CCHF Double Antigen Multi-species ELISA kit (Innovative.Diagnostics, France).

#### Molecular methods

We extracted RNA from clinical samples using the QIAamp Viral RNA Kit (Qiagen, Germany). For the detection of CCHFV, we applied two real-time RT-PCR assays: Congo-Crimea Real-TM (Sacace Biotechnologies Sri, Italy) and CCHFV RT-qPCR (Bio-Speedy, Bioeksen, Türkiye). We further analysed the positive samples using RT-nested PCR which amplified a 260 bp fragment of the S RNA segment of the virus [11]. The PCR products were Sanger sequenced and analysed using the BLAST tool from the National Center for Biotechnology Information. For RNA extraction from the animal and tick samples, we used the QIAamp cador Pathogen Mini Kit (Qiagen) followed by real-time RT-PCR using the CCHFV RT-qPCR (Bio-Speedy, Bioeksen).

Next generation sequencing (NGS) of the RNA extracted from the blood of the index case, was done on an Ion Torrent S5 platform (Thermo Fisher Scientific, United States). Raw reads were processed through the Torrent Suite Software for quality control, and the sequences were mapped using as reference the CCHFV sequences of Kuchica/North Macedonia-1/2023 (GenBank Accession numbers PP729064, PP729065, PP729066 for the S, M and L segments, respectively). Assembly and annotation of the CCHFV whole genome sequences were performed using Geneious version 7.1.3. We performed the phylogenetic analysis in MEGA version 12 software [12].

#### Virus isolation

We made no attempts at virus isolation to avoid any contamination.

### Serosurvey

To assess the infection prevalence and transmission risk in the affected area, we conducted a serosurvey between 4 and 23 July among volunteers recruited from residents of villages within a radius of less than 6 km from the case’s residence) and among volunteers from the broader area (radius of 6–10 km). The recruitment of volunteers was carried out through a communication campaign that included leaflet distribution, announcements in local media, and the involvement of local community leaders. Serum samples were collected, and all participants completed a questionnaire on demographic characteristics (age, sex, place of residence), occupational characteristics, educational level and potential exposures. These included contact with animals or participation in activities associated with tick or animal exposure (e.g. hiking, hunting, camping), as well as history of confirmed tick bites or animal exposure and their timing.

### Statistical analysis

The analyses were performed using IBM SPSS Statistics 29.0. We initially summarised the primary variables of the study using descriptive statistics. Continuous variables are reported as mean and standard deviation for normally distributed data, and as median and interquartile range for non-normally distributed data. We assessed differences in seroprevalence proportions between groups using the chi-squared test or Fisher’s exact test, as appropriate, with corresponding 95% confidence intervals (CI). All tests were two-tailed and a p value < 0.05 was considered statistically significant.

## Results

### Case 1

Serial laboratory results of Case 1 are described in Table 1. 

**Table 1:** Laboratory parameters of Crimean Congo haemorrhagic fever Case 1, Greece, 2025

Parameter	Day 1 (admission)	Day 2	Day 3	Day 4	Day 5	Day 6	Day 7 (death)
Hb g/dL (normal range: 12-18 g/dL)	10	9.2	7.7	5.8	8.2	8.7	8.6
WBC/μL (normal range: 4,000–11,000/μL)	6,700	5,000	4,800	9,000	5,300	3,100	2,200
PLT/μL (normal range: 150,000–450,000/μL)	20,000	7,000	6,000	33,000	68,000	81,000	39,000
INR (ULN: 1.15)	1.84	2.23	2.16	2.38	2.41	3.25	NA
aPTT (ULN: 36 sec)	49	81	73	67	69	65	NA
Fibrinogen (normal range: 180–400 mg/dL)	NA	83	120	68	65	NA	NA
Ferritin (ULN: 400 ng/mL)	26,791	89,900	105,707	98,390	162,820	162,420	141,560
Urea (ULN: 48 mg/dL)	112	154	208	92	51	30	16
Creatinine (ULN: 1.2 mg/dL)	2.23	3.46	4.9	2.73	1.92	1.77	1.75
LDH (ULN: 224 U/L)	895	2,455	3,951	7,352	21,792	19,106	16,177
AST (ULN: 40 U/L)	280	938	2,384	5,928	23,090	21,229	17,453
ALT (ULN: 40 U/L)	87	269	775	1,638	5,046	3,802	2,438
ALP (ULN: 130 U/L)	97	170	223	221	296	336	449
Bil (ULN: 1.2 mg/dL)	0.7	0.85	1.88	2.16	2.42	3.1	3.75
γGT (ULN: 61 U/L)	126	181	200	111	110	142	154
Triglycerides (ULN: 150 mg/dL)	98	96	92	188	286	445	963

ALP: alkaline phosphatase; ALT: alanine aminotransferase; aPTT: activated partial thromboplastin time; AST: aspartate aminotransferase; Bil: bilirubin; γGT: gamma-glutamyl transferase; Hb: haemoglobin; INR: international normalised ratio; LDH: lactate dehydrogenase; NA: not available; PLT: platelets; ULN: upper limit of normal; WBC: white blood cells.

During hospitalisation, blood and fresh frozen plasma transfusions along with fibrinogen replacement therapy were also given. Despite aggressive supportive measures with corticosteroids and intravenous γ-immunoglobulin (IVIG), and escalation of antimicrobial treatment, the patient continued to worsen and was subsequently transferred to the intensive care unit on day 6, where they developed multi-organ failure and diffuse lung and gastrointestinal haemorrhage. 

All blood and urine cultures, and serological testing for *Leptospira*, *Leishmania, Brucella* and hantaviruses were negative. However, CCHFV (genus Orthonairovirus, Family *Nairoviridae*) was detected by real-time RT-PCR and by conventional RT-PCR in the patient’s blood sample which was sent to the NRC on day 9 from symptom onset (day 7 of admission), with a quantification cycle (Cq) value of 8.29. On the same day, the testing by ELISA for CCHFV IgM and IgG antibodies resulted negative. The NRC notified clinicians and the NPHO of the positive result. Notably, CCHFV IgM and IgG antibodies were not detected in subsequent serum samples of the index case (taken on days 5, 7 and 9 of disease). The patient died on day 9 from symptom onset.

### Epidemiological investigation

Immediate response was undertaken, including a thorough case investigation, to identify the likely mode and place of infection and identify potential risk factors. During the incubation period, the patient had not visited another area beyond his village and a nearby livestock farm, in the same municipality. No tick bite was reported, nor contact with blood or body fluids from animals.

We performed contact tracing in the household and in the healthcare facilities that the index patient had visited while viraemic, including the hospital, one primary healthcare centre and a private physician’s practice. Contacts were categorised according to the risk of their exposure (Box 1), and health status self-monitoring was recommended for 14 days after their last contact with the patient. Samples from all contacts who developed any compatible symptoms (i.e. fever or symptoms of viral syndrome) during this period were sent to the NRC for testing. Contact tracing of Case 1 revealed 82 contacts within the hospital (of whom 17 were classified as high risk), as well as five household contacts and six contacts in the other healthcare settings (all classified as low risk). A total of 10 contacts, including four who developed mild symptoms (fever and/or myalgia and/or arthralgia), were tested for CCHF; nine had negative results in PCR and IgM serology, and one contact tested positive (Case 2).

Box 1 **Contact risk categorization criteria, Crimean Congo haemorrhagic fever, Greece, 2025**

**High-risk contact: **A person directly or indirectly exposed to blood, bodily fluids or tissues of a viraemic patient while not wearing personal protective equipment.

**Low-risk contact:** A person directly or indirectly exposed to blood, bodily fluids or tissues of a viraemic patient while wearing personal protective equipment.

### Case 2

The secondary Case 2 was diagnosed 2 days after the diagnosis of Case 1. That patient was a middle-aged physician with no underlying medical conditions who had been on duty during the index patient's admission to hospital and had provided direct care on the first 2 days of their hospitalisation. The physician reported contact with the index case while wearing gloves. Case 2 reported fever (38.5 °C), myalgia and sore throat 5 days after the last contact with Case 1. Laboratory investigation showed leukopenia, thrombocytopenia, elevated levels of LDH, d-dimers, triglycerides and ferritin (Table 2).

**Table 2:** Laboratory parameters of Crimean Congo haemorrhagic fever Case 2, Greece, 2025

Parameter	Day 1 (admission)	Day 3	Day 4	Day 5	Day 7 (discharge)
Hb g/dL(normal range: 12-18 g/dL)	12.2	11.91	2.4	12.1	11
WBC/μL(normal range: 4,000–11,000/μL)	1,500	4,300	3,900	3,400	4,800
PLT/μL(normal range: 150,000–450,000/μL)	80,000	110,000	105,000	140,000	175,000
INR (ULN: 1.15)	0.98	0.76	0.73	0.82	0.86
aPTT (ULN: 36 sec)	37	28	26	25	22
Fibrinogen (normal range: 180–400 mg/dL)	290	237	216	220	223
D-dimers (ULN: 243 ng/mL)	1,250	697	739	795	551
Urea (ULN: 48 mg/dL)	28	28	28	34	40
Creatinine (ULN: 1.2 mg/dL)	1.02	0.87	0.87	0.84	0.8
LDH (ULN: 224 U/L)	310	287	292	293	308
AST (ULN: 40 U/L)	48	38	54	65	45
ALT (ULN: 40 U/L)	21	20	33	46	42
ALP (ULN: 130 U/L)	94	71	72	72	73
Bil (ULN: 1.2 mg/dL)	0.38	0.44	0.69	1.44	1.8
γGT (ULN: 61 U/L)	22	33	62	83	90
Ferritin (ULN: 400 ng/mL)	1,077	762	692	819	989
Triglycerides (ULN: 150 mg/dL)	170	197	205	140	135

ALP: alkaline phosphatase; ALT: alanine aminotransferase; aPTT: activated partial thromboplastin time; AST: aspartate aminotransferase; Bil: bilirubin; γGT: gamma-glutamyl transferase; Hb: haemoglobin; INR: international normalised ratio; LDH: lactate dehydrogenase; PLT: platelet; ULN: upper limit of normal; WBC: white blood cell.

Because of the recent contact with the index case, a blood sample was immediately sent to the NRC, where CCHFV infection was confirmed by real-time RT-PCR (Cq = 16.75). The patient was already in self-isolation, as recommended to the index patient’s symptomatic contacts. Upon receipt of the positive result, the patient was admitted to the infectious diseases unit of the Department of Medicine, and treated with oral ribavirin as per World Health Organization recommendations (30 mg/kg initial dose, then 15 mg/kg every 6 h for 4 days, then 7.5 mg/kg every 8 h for 6 days) [13], along with corticosteroids (one pulse intravenous infusion of 500 mg methylprednisolone followed by 1 mg/kg/day of prednisolone with appropriate rapid tapering) and IVIG (0.4 g/kg/day for 5 days). The patient gradually improved during the following days and was discharged on day 9 after symptom onset, with full recovery, even though anaemia was recorded because of ribavirin use, which was treated successfully with subcutaneous erythropoietin. RT-PCR testing remained positive on day 9 (Cq = 25.33) and day 17 (Cq = 28.30). On the same 2 days, RT-PCR was still positive at Cq = 25.33 and Cq = 28.30, respectively. Notably, CCHFV-specific IgM was not detected in the serum sample taken on day 2, while it was detected in the serum sample taken on day 9.

Contact tracing of the secondary case identified one low-risk household contact and one hospital contact (of low risk). Therefore, no testing was performed. 

### Molecular findings: phylogenetic analysis

**Figure 1:**
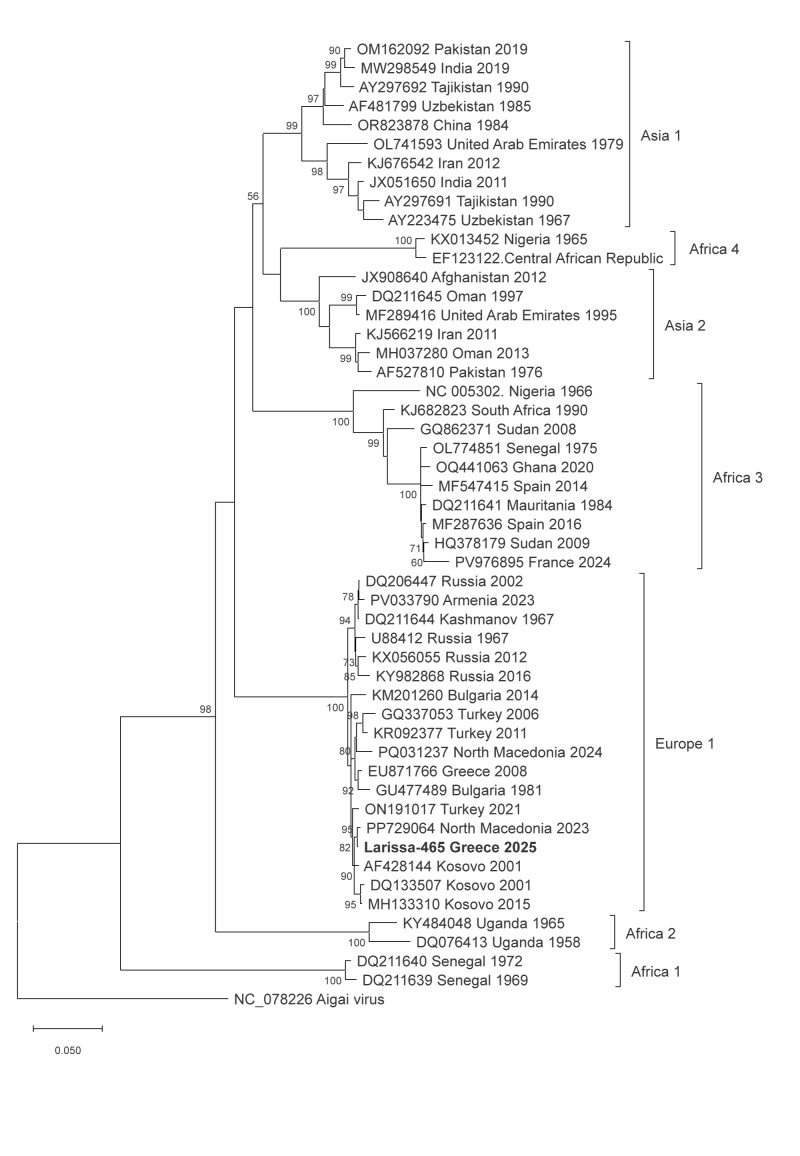
Maximum likelihood phylogenetic tree, Crimean Congo haemorrhagic fever virus S RNA segment encoding the nucleocapsid protein (1,446 nt), index case, Greece, 2025

**Figure 2:**
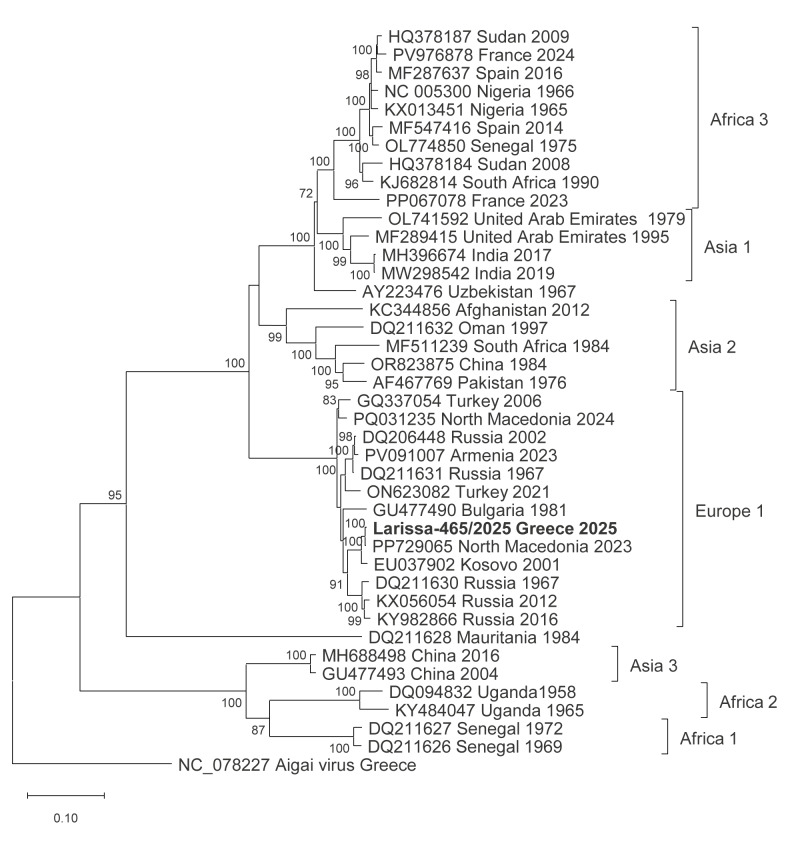
Maximum likelihood phylogenetic tree, Crimean Congo haemorrhagic fever virus M RNA segment encoding the glycoprotein precursor (4,302 nt), index case, Greece, 2025

**Figure 3:**
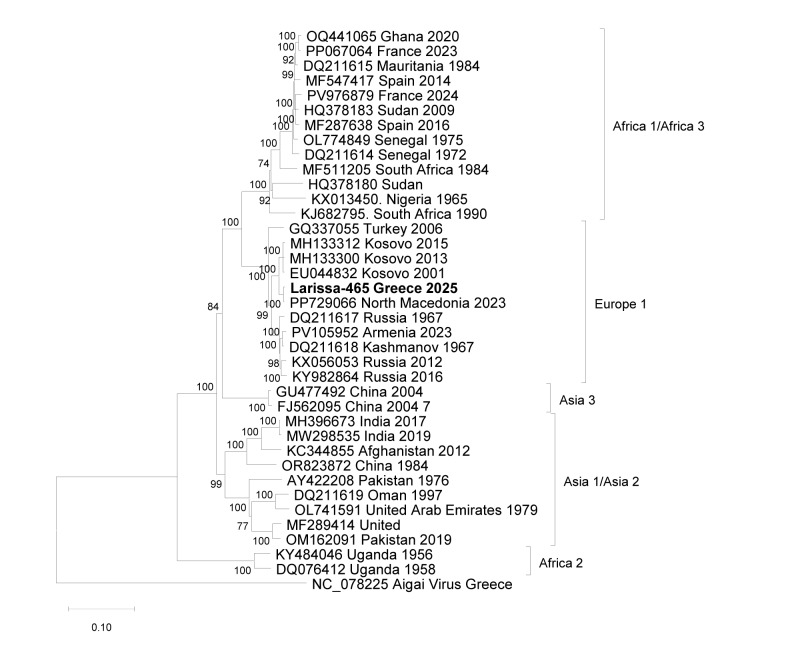
Maximum likelihood phylogenetic tree, Crimean Congo haemorrhagic fever virus L RNA segment encoding the RNA-dependent RNA polymerase (11,835 nt), index case, Greece, 2025

Sanger dideoxy sequencing of the PCR product from the index case indicated that the sequence clustered into CCHFV lineage Europe 1 (genotype V) and showed 99% identity with sequences from other Balkan countries. Whole genome sequences of all three CCHFV genome segments (small (S), medium (M) and large (L) were used to prepare phylogenetic trees based on the complete open reading frames of the three segments (Figures 1, 2 and 3). In all phylogenies, the sequences of the present study clustered together with the respective sequences of the strain Kuchica/North Macedonia-1/2023.

The percentage of replicate trees in which the associated taxa clustered together (1,000 replicates) is shown next to the branches; only values > 70% are shown. The evolutionary rate differences among sites were modelled using a discrete gamma distribution across five categories (+G, parameter = 1.3285), with 52.82% of sites deemed evolutionarily invariant (+I). The sequence of the present study is shown in bold. Sequences are shown as accession number, country and year of detection. Evolutionary analyses were conducted in MEGA12.

The percentage of replicate trees in which the associated taxa clustered together (1,000 replicates) is shown next to the branches; only values > 70% are shown. The evolutionary rate differences among sites were modelled using a discrete gamma distribution across five categories (+G, parameter = 1.7727), with 44.18% of sites deemed evolutionarily invariant (+I). The sequence of the present study is shown in bold. Sequences are shown as accession number, country and year of detection. Evolutionary analyses were conducted in MEGA12.

The percentage of replicate trees in which the associated taxa clustered together (1,000 replicates) is shown next to the branches; only values > 70% are shown. The evolutionary rate differences among sites were modelled using a discrete gamma distribution across five categories (+G, parameter = 2.8867), with 55.61% of sites deemed evolutionarily invariant (+I). The sequence of the present study is shown in bold. Sequences are shown as accession number, country and year of detection. Evolutionary analyses were conducted in MEGA12.

### Epizootiological and environmental investigations

Field investigation was conducted 2 days following the diagnosis of Case 1. Blood samples were collected from 10% (n = 50) of the farm animals (sheep), and 150 animals were thoroughly screened for ticks. Overall, we collected 11 ticks feeding on the animals; seven were identified as *Hyalomma marginatum* (the main CCHFV vector in Europe) and four as *Rhipicephalus bursa*. In addition, three *Hyalomma aegyptium* ticks were collected from a turtle in the broader area. We also performed flagging for ticks in the area of the farm (500 m^2^); however, no ticks were found. Ticks and animal blood samples resulted negative for CCHFV RNA, and the serological testing of the animals’ blood samples was also negative.

### Serosurvey

We collected a total of 128 samples. Among the participants, 55% (n = 70) were male and 45% (n = 58) female. Overall, CCHFV IgG antibodies were detected in six (4.7%; 95% CI: 1.6–8.6) of serum samples; CCHFV IgM antibodies were not detected. The mean age of seropositive individuals was significantly higher compared with seronegative individuals (83.0 vs 58.7 years, p < 0.001). The seropositive samples were from six among the 87 residents sampled in the villages nearest the index case’s residence (within a 6 km radius), while no positives were detected among the 41 samples from more distant areas (6.9% vs 0%, p = 0.176). In addition, individuals with education at the basic to high school level had a significantly higher likelihood of seropositivity compared with those with education above high school (8.7% vs 0%, p < 0.02).

## Outbreak control measures

The One Health response consisted of a series of simultaneous measures, implemented with the collaboration of stakeholders from the public health, healthcare and animal health sectors, at national and regional levels. The NPHO/Ministry of Health and the Ministry of Rural Development and Food (MRDF, national veterinary authorities) coordinated the response. The day after Case 1’s diagnosis, an urgent multi-sectoral meeting of all stakeholders from all involved sectors (national and regional public health and veterinary authorities, NRC, regional government, university hospital, experts) was convened to coordinate the response.

### Infection prevention and control measures

Case 1 was immediately isolated upon CCHF suspicion on day 6 of hospitalisation. Immediate notification and collaboration with the hospital’s infection control committee enabled the rapid implementation of measures to prevent further nosocomial transmission, including contact tracing and monitoring of contacts for 14 days. The need for strict adherence to infection control procedures was underlined to all healthcare professionals of the local healthcare units. Appropriate procedures were followed for the safe management of the corpse.

### Contact management

Following notification of the index case, the NPHO immediately supplied oral ribavirin from its stock pharmacy for post-exposure prophylaxis in high-risk contacts. In addition to the secondary case who received ribavirin for treatment, ribavirin was recommended for prophylaxis to a further seven high-risk contacts of the index case; however, only one contact accepted prophylaxis, which was taken for a few days until a negative PCR test was obtained.

### Prevention and control measures for vectors and animals

Targeted environmental vector and rodent control measures were recommended in and around the farm and the index patient’s household. In parallel, the national veterinary authorities (MRDF) recommended targeted prevention and control measures for the specific farm’s animals, including blood and tick sampling, restriction of animal movements from the farm, epizootiological investigation in order to determine possible sources of infection, intensified disinfestation protocols for the livestock and tick control. The local veterinary authority of the Regional Unit where the related farm was located was urgently informed, in order to conduct an on-site visit and apply necessary measures.

Nationwide guidance was also issued to veterinary services to enhance awareness and inform animal breeders and relevant stakeholders about mandatory animal disinfestation, reinforcement of biosecurity measures on animal farms, in slaughterhouses and during animal transport. In addition, a nationwide awareness campaign was recommended targeting high-risk groups (professionals handling animals or biological materials, or potentially at risk of contact with ticks) and promoting the adoption of personal protection and hygiene measures.

### Communication

Recognising that raising awareness is critical in preventing the infection and limiting further transmission, NPHO and MRDF undertook communication activities to inform and engage all relevant professionals and the public (Box 2).

Box 2 **Public health communication in response to the recording of Crimean Congo haemorrhagic fever cases, Greece, 2025**

- A press release was issued 2 days after the diagnosis of Case 1 to inform the public and high-risk groups.
- The local public healthcare facilities were urgently informed about the index case and about the need for vigilance for other suspected cases and for strict adherence to infection control procedures.
- An informative letter was disseminated to healthcare professionals nationwide 9 days after the diagnosis of Case 1 (including a case definition for suspected cases).
- An in-person informative meeting was conducted at the local primary health centre 8 days after the diagnosis of Case 1.
- A training webinar for healthcare professionals was conducted a month later.
- A communication campaign targeting the local population and high-risk groups was developed, including public meetings held in two villages in the affected area within a week of Case 1’s diagnosis (including the distribution of informational leaflets in the broader region).
- A circular was issued and sent 2 days after the diagnosis of Case 1 to all veterinary authorities in the country, providing information, guidelines and measures for involved livestock professionals and stakeholders, as well as specific measures for the related farm.
- An informative leaflet was issued, uploaded on the MRDF website and sent to all veterinary authorities 1 month after Case 1’s diagnosis, with the aim to be disseminated to involved livestock stakeholders.
- The NPHO timely reported both CCHF cases to the European Centre for Disease Prevention and Control and informed the Health Security Committee, the European Emerging and Vector-borne Diseases network and the World Health Organization through the Early Warning and Response System (EWRS) and the EpiPulse portal.

## Discussion

To date, three autochthonous CCHF cases have been recorded in Greece, namely the two cases described here, and another one reported in 2008 in Thrace region, northern Greece, with a fatal outcome [5]. Although CCHF is not considered endemic in Greece, the disease is endemic in neighbouring countries. A case imported from Bulgaria was reported in 2018, emphasising that both autochthonous infections and travel-associated cases should be considered [14,15].

Given previous surveillance data and the distance from the borders with endemic countries, the occurrence of this recent event in central Greece was quite unexpected. The exact importation route of CCHFV remains unclear; taking into account the phylogenetic results, introduction from neighbouring endemic countries is suspected, probably via small mammals or migratory birds, or even through animal trade; migratory birds and movement of domestic or wild animals from endemic countries facilitate the introduction of *Hyalomma* ticks or viraemic animal hosts [16,17], which is a potential risk for cross-border spread.

Ticks of the genus *Hyalomma* are present in Greece, albeit at very low rates compared with other dominant tick species (e.g. *Rhipicephalus* spp.) [18]. This is also supported by unpublished data from an ongoing extensive national-wide tick sampling project in the context of the European OH SURVector project, where, in 2024, only 1% (n = 25) of the 2,193 collected ticks belonged to the *Hy. marginatum* species. Moreover, none of the ticks collected in 2024 tested positive for CCHFV. The field investigations, timely conducted as part of the public health response, resulted in the collection of a few *Hy. marginatum*, revealing the presence of this species in the area. Interestingly, all ticks and animals tested from the farm were CCHFV-negative. Thus, we do not have evidence that this farm was the source of infection. However, further studies are needed in both ticks and animals to elucidate the exact distribution of the virus. Also, it should be noted that wildlife and related ticks were not investigated.

We detected CCHFV IgG antibodies in 4.7% of humans tested. Previous seroprevalence studies in Greece showed CCHFV IgG antibodies in 4% of human population [18]. It has to be mentioned that another orthonairovirus, Aigai virus, is present in Greece (and in other Balkan countries), which cross-reacts with CCHFV serological tests, therefore the discrimination in serology is not possible [19]. There is a need for specific laboratory assays capable of distinguishing the two viruses in serology.

Notably, all seropositive cases were clustered in the nearest villages around the index case, supporting localised transmission dynamics, although the small sample size limits firm conclusions. In addition, the association between lower education levels and seropositivity could reflect differences in occupational exposure, awareness or preventive practices.

These two recent CCHF cases in Greece reinforce the main dual transmission routes of the virus: zoonotic transmission from infected ticks to humans, and subsequent nosocomial spread through healthcare exposure [20]. The index case had all the relevant prognostic factors associated with a severe course of the disease such as somnolence, bleeding, severe thrombocytopenia, elevated liver enzymes and overt disseminated intravascular coagulation because of severe secondary haemophagocytic lymphohistiocytosis syndrome (HLS) even though he received adequate HLS treatment; furthermore, the high viral load is the strongest predictor for adverse outcome [20].

When CCHFV infection is suspected or confirmed, all healthcare workers in contact with the patients should use appropriate PPE [21]. The occurrence of the secondary case highlights the importance of meticulous and continuous adherence to protective measures. Although the physician reported wearing gloves, a potential exposure may have occurred during repeated glove changes while managing a severe bleeding episode of the index case.

The CCHFV infections were promptly confirmed at the NRC, underscoring the importance of specialised diagnostic capacity [22]. Although the effectiveness of ribavirin in CCHF is not firmly evidence-based, and we cannot conclusively attribute the recovery of our secondary case to its use, the prompt initiation of ribavirin together with supportive care may have contributed [13,23]. In addition, the fact that this was a secondary case could potentially explain the favourable outcome, as some data suggest that secondary cases can be milder with lower viral loads [24]. As an example, a case series of secondary CCHF among healthcare workers in Türkiye reported a case fatality rate (CFR) of 11.1% [6], while another case series from Iran reported a CFR of 16.7% among secondary cases [25]. Nevertheless, the evidence remains conflicting, as nosocomial and secondary transmissions have also been associated with severe and even fatal outcomes with a CFRs exceeding 30% [26,27].

Even though the mechanisms underlying thrombocytopenia, a hallmark of CCHF, remain poorly understood, it was decided to manage Case 2 in a double targeting way against active viral replication (ribavirin) but also against a potential underline component of virus-induced immune thrombocytopenia and a virus-induced threatening HLS (steroids plus IVIG) because of high ferritin levels, cytopenia, fever and hypertriglyceridaemia.

## Conclusions

Climate change is expected to affect the life cycle of arthropods, including ticks, potentially increasing their abundance and geographical distribution. Enhanced awareness of CCHF among clinicians and public and animal health professionals is critical. Clinicians should maintain a high index of suspicion in when receiving febrile patients with thrombocytopenia and haemorrhagic signs even in non-endemic areas. Rigorous IPC, including full barrier PPE when managing suspected cases, is of utmost importance to mitigate further nosocomial spreading. The effectiveness of ribavirin as potential CCHF treatment and/or as prophylaxis, as well as of the management of subsequent virus-induced HLS should be further validated, based on evidence, while there is an urgent need for specific drugs and effective vaccines. The recent recording of autochthonous CCHF cases in Greece highlights the need for sustained vigilance and prompt coordinated response across the public, animal and environmental health sectors. In line with the One Health approach, sustained intersectoral collaboration, enhanced surveillance, awareness of high-risk groups, and prompt field investigations are critical to assess and mitigate the risk and ensure rapid response to any future events.

## Data Availability

Data related to the cases (clinical information, laboratory results, serosurvey) will be available from the corresponding author upon request. The sequences of the present study were submitted to the GenBank Database and received the Accession numbers PX499073-PX499075.
